# Global health inequities: more challenges, some solutions

**DOI:** 10.2471/BLT.24.291326

**Published:** 2024-02-01

**Authors:** Viroj Tangcharoensathien, Angkana Lekagul, Yik-Ying Teo

**Affiliations:** a International Health Policy Program, Ministry of Public Health, Tivanond Road, Nonthaburi, 11000, Thailand.; b Saw Swee Hock School of Public Health, National University of Singapore, Singapore.

Health inequity is the presence of unfair, avoidable or remediable differences in achieving optimal health and well-being among people. However, despite global commitment to reduce health inequities, progress has been uneven^1^ or even delayed by slow progress towards universal health coverage (UHC).^2^ The current geopolitical tensions and high number of refugees further compound the challenges of reducing health inequities.^3^

This theme issue of the *Bulletin of the World Health Organization* shows how health inequities affect many areas, both at national and global scale. The papers discuss health inequity and its root causes, and offer promising solutions. Challenges include national statistics not capturing health inequity among vulnerable populations such as Indigenous people, refugees and migrant workers,^4^ including migrant health workforce.^5^ However, good practices exist, with one paper describing^6^ how in Australia local Indigenous communities successfully manage primary health-care clinics. With an empowered Indigenous population and continued government support, these practices can be scaled up and replicated. Another paper describes the World Health Organization (WHO) guidelines on self-care, a promising intervention to access hard-to-reach populations in efforts to achieve UHC.^7^

Monitoring the trends of health inequities in countries is important to guide policies. One index that could be suitable for evaluating equity changes over time in low- and middle-income countries is the socioeconomic deprivation index. In this issue, a study shows how the index, based on data from routine household surveys, can be used to analyse the coverage of maternal and child interventions by socioeconomic deprivation status.^8^

A subtle and invisible type of inequity is uncovered in a study revealing that independent evaluations of global health initiatives are often undertaken by consulting firms or consultants from high-income countries.^9^ These experts often have limited knowledge and understanding of the sociopolitical, cultural and health system contexts of countries, and yet produce policy recommendations based on their assessments. The authors of this study provide suggestions on how to rectify this inequity, though further impact assessments are needed.

Another paper suggests that decolonization processes need to address the root causes of the power imbalance between high- and low-income countries.^10^ Contemporary forms of colonialism, notably corporate and financialized colonialism through globalized systems of wealth extraction and profiteering, lead to inequitable global health systems. Recruitment of health personnel from low- and middle-income countries to high-income countries can be categorized as a form of neo-colonization. The WHO *Global code of practice on international recruitment of health personnel*^11^ offers guidance in this respect. Although more information is needed to assess the Code’s effectiveness, one of its gaps is insufficient level of implementation to realize its full potential towards UHC and reducing health inequity.^12^

In the ongoing negotiations for the amendment of the International Health Regulations 2005 (IHR), and for a Pandemic Agreement, both of which are expected to be concluded and adopted by the World Health Assembly in May 2024, a member of the IHR Review Committee provides insights on the complex landscape of global health security.^13^ WHO’s authority in global health security is eroding, and high-income countries use forum shifting to consolidate their positions. Forum shifting, defined as a strategy to influence negotiations towards texts that better meet actors’ needs, is illustrated by high-income countries arguing at the Intergovernmental Negotiating Body that the waiver of intellectual property be discussed at the World Trade Organization (WTO). Their stance remained unchanged although the WTO Ministerial Conference had decided on Trade-Related Aspects of Intellectual Property Rights waivers for the coronavirus disease 2019 (COVID-19) vaccine on 17 June 2022.^14^ A different example of negotiation strategy is the Group for Equity that negotiated the Pandemic Agreement on behalf of low- and middle-income countries from the WHO African Region and other like-minded countries.^13^

Another article published in this issue reports on an increasing trend of including corporate social responsibility provisions in international investment agreements.^15^ Despite varied detail of commitment, these provisions offer a potential tool to increase government guidance and the accountability of global corporations, including with respect to public health commitments. Such is the case with provisions that require investors operating in the host country to refrain from seeking exemptions to health policies. Furthermore, the United Nations Global Compact, a voluntary corporate sustainability initiative, encourages alignment of corporate strategies and operations with principles regarding human rights, labour, environment and anti-corruption. Companies adopting the initiative show increased sales growth and profitability, though no difference in labour productivity.^16^

This theme issue of the Bulletin was launched at the Prince Mahidol Award Conference 2024. Rectifying global health inequity requires multidimensional interventions and decisive government leadership at the macro-policy level, collaboration with affected populations at the micro-operational level and accelerating progress towards UHC. Strengthening aid effectiveness by recognizing countries’ priorities^17^ and regular monitoring of health inequities to guide policy are needed.
